# The pitfalls of focusing on cardiovascular disease mortality to explain differences in life expectancy

**DOI:** 10.1007/s10654-023-01089-y

**Published:** 2024-01-10

**Authors:** Susanne Stolpe, Bernd Kowall, Andreas Stang

**Affiliations:** 1 Institute for Medical Informatics, Biometry and Epidemiology, University Hospital Essen, Essen, Germany; 2 Department of Epidemiology, School of Public Health, Boston University, Boston, USA

In their essay, Jasilionis et al. claim that the relatively low life expectancy in Germany is mainly due to higher CVD mortality [1]. They conclude that substantial problems in prevention of modifiable cardiovascular risk factors are the main reason for “The underwhelming life-expectancy of Germany”. Unfortunately, the authors do not present examples for best practice in prevention in the selected countries with higher life expectancy.

We would like to address three points to consider when discussing life expectancy and CVD mortality.

## Impact of quality of cause of death statistics

**Fig. 1:**
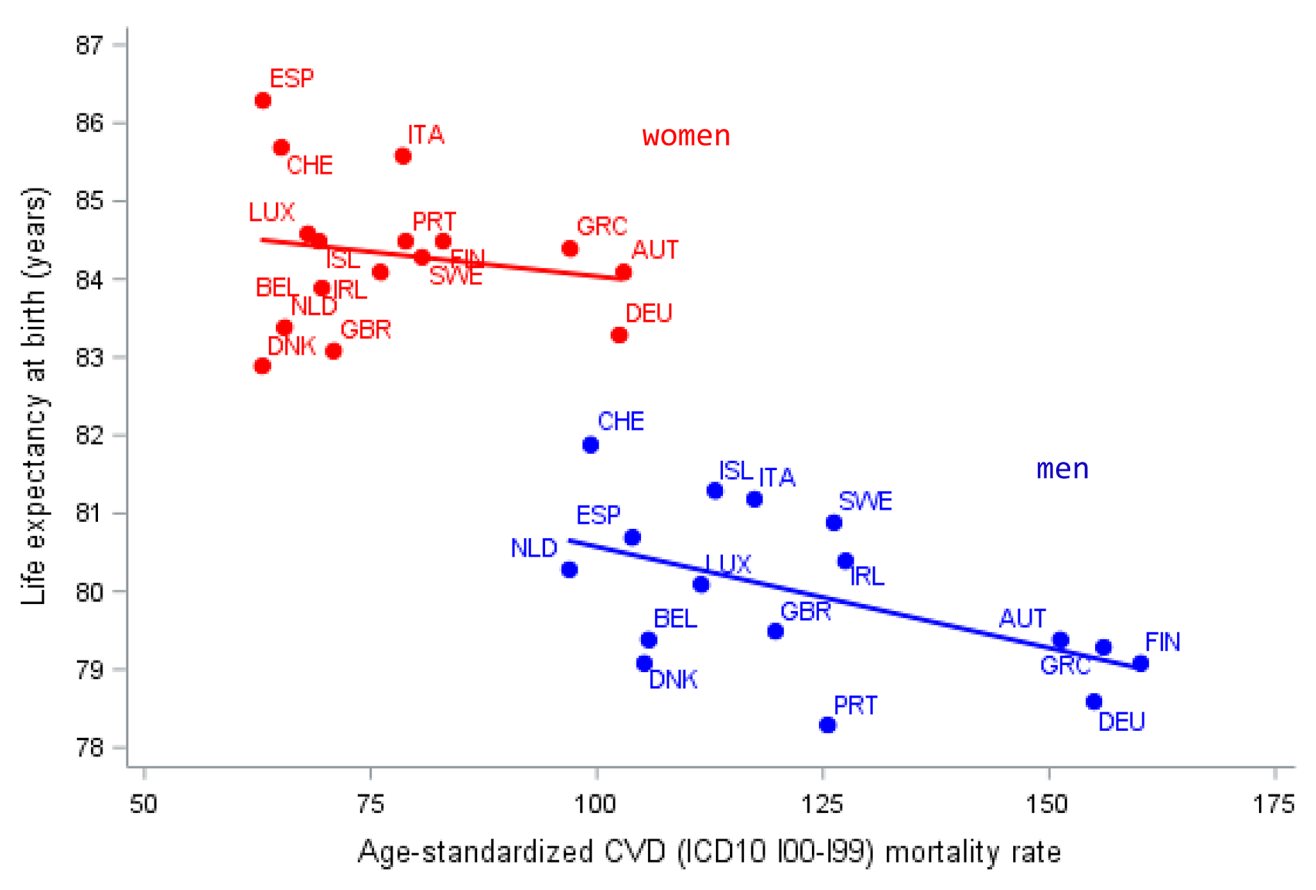
Association between age-standardized CVD mortality and life expectancy 2018. (Data source: stats.oecd.org)

First, we doubt that reported age-standardized CVD mortality rates are closely associated with a country’s life expectancy. For Western European countries, we fitted a linear regression model for the association between CVD mortality rates and life expectancy. For 2018, the adjusted R² was -0.04 for women and 0.23 for men (Fig. 1).

The main reasons why CVD mortality rates cannot explain much of the variability in life expectancy in Western Europe is that data on cardiovascular causes of death (CoD) are only comparable to a limited extent between countries and that these rates do not necessarily mirror the disease burden of a population [2].

This can be illustrated comparing reported age-standardized CVD mortality of Germany, Austria, Denmark and the Netherlands for 2018: the respective rates were 127, 125, 82 and 80 per 100.000. These differences are hardly explainable by differences in population characteristics, morbidity or by health care quality.

CoD statistics comprise data that result from individual decisions by physicians who certify death and who decide which disease(s) among others– in a probably multi-morbid person - to enter on a death certificate. The selected CoD of a deceased person with the same medical history, sex and age will often differ in Germany, Spain, France or elsewhere. Factors influencing the selection of a CoD are manifold and relate – among others – to time and place of certification, available patient information and a physician’s experience and knowledge of WHO rules of regarding CoD.

The non-standardized collection of CoD data affects mortality statistics three-fold:

- Less trained physicians may more often select CoD that do not meet the WHO definition of CoD (so called garbage codes). The larger the proportion of garbage-coded deaths, the lower the quality of mortality statistics and the greater a possible distortion when CoD data is analysed. The frequency of garbage-coded deaths differs by country, year, and age and sex of a deceased, and can reach up to 30% in Western European countries [3].
- National preferences influence the probability of selecting a specific CoD. Especially in deaths of multimorbid persons, several potential starting points for the causal chain leading to death are imaginable. The decision which starting point to select as underlying CoD is triggered by non-clinical factors such as personal or national preferences or the awareness for a specific CoD (e.g. dementia) [2]. This is visible when comparing the proportions of CVD deaths among all deaths in 2018 in Germany (39%), Austria (42%), Denmark (21%) and the Netherlands (25%) – countries that are comparable in population and health system quality.
- In general, the accuracy of CoD certification is known to be problematic. It can only be determined comparing autopsy data, which has been rarely done [4]. Especially the validity of CVD as CoD can be assumed as being low [4]. Comparable data on CoD validity in Europe is missing.

## Choice of country and outcome

Second, comparing healthy life expectancy as outcome, it decreased in many Western European countries such as NL, DK, AU or UK from 2008 to 2016, but increased most in Germany. In the healthy life expectancy ranking, Germany improved from rank 23 to 6 among 27 EU countries for women and from 22 to 7 in men [5]. The inclination to rate one’s health as ‘good or very good’ may differ between countries and affect the prevalence of self-rated ‘good or very good health’ and therefore healthy life expectancy [6].

The authors did not include Denmark whose life expectancy is comparable with Germany (2019: 81.3 years in Germany, and 81.5 years in Denmark), and which is often presented as role model in Germany for successful restructuring health care due to favourable CVD outcome indicators [https://www.n-tv.de/panorama/Krankenhausreform-in-Deutschland-Was-wir-von-Daenemark-lernen-koennen-article23773989.html, accessed 30.10.2023].

## Relevance of non-health-care related factors affecting life expectancy

Third, we think, non-health related factors (attitudes, culture, values) that constitute a population should receive more attention when discussing life expectancy. While the structures, performance and efficiency of Western European health care systems changed considerably in recent decades, Germany’s low ranking in life expectancy has been stable for about 30 years. This suggests that there are factors acting in the background, a nation´s ‘DNA’ or core, which remain quite stable over decades and influence life expectancy.

In the more recent literature, gross domestic product per capita, education, pension levels, but also a country’s culture have been associated with life expectancy. The grade of individuality in a society, its long or short-term orientation, gender, and socio-economic equality were described as possibly influential factors for life expectancy [7]. Very recently, using individual cohort data in the USA, after 25 years follow-up, a higher social network score was strongly associated with higher life expectancy – independent of age or frailty [8].

It is conceivable that the core, the “character” of a nation influences the response to disease and that this core builds the base for a population’s life expectancy, which then can be further increased by public health measures. Possibly, close family ties and regular contacts with friends and neighbours affect life expectancy more than high-tech health care offers.

This might explain roughly why a country’s rank in life expectancy was quite stable over time – and why life expectancy is increasing rather simultaneously in Western Europe – as health care progresses universally.

## Conclusion

Life expectancy is markedly influenced by factors not susceptible to health policy. These are rarely taken into account in analyses on the effect of cardiovascular mortality, for which international comparisons are complicated. Researchers and policy makers should be cautious using CoD statistics to compare CVD mortality – or mortality from specific cardiovascular causes - between countries and over time to evaluate the effectivity of health care and health policy interventions. Recoding of garbage coded deaths should be mandatory [9]. Standardized quality reviews of CoD certification and regular training of physicians can increase the usability of mortality data [10]. These measures, however, do not reduce the problems arising from selecting a CoD in multi-morbid persons or the very old Research on influencing factors for life expectancy should include sociological and socio-political factors as well.
